# Identification and Validation of Autophagy-Related Genes in Primary Ovarian Insufficiency by Gene Expression Profile and Bioinformatic Analysis

**DOI:** 10.1155/2022/9042380

**Published:** 2022-07-04

**Authors:** Siji Lv, Jiani Sun, Jing Sun

**Affiliations:** Department of Gynecology, Shanghai First Maternity and Infant Hospital, School of Medicine, Tongji University, Shanghai, China

## Abstract

**Background:**

To investigate the relationship between primary ovarian insufficiency and autophagy, we detected and got the expression profile of human granulosa cell line SVOG, which was with or without LPS induced. The expression profile was analyzed with the focus on the autophagy genes, among which hub genes were identified.

**Results:**

Totally, 6 genes were selected as candidate hub genes which might correlate with the process of primary ovarian insufficiency. The expression of hub genes was then validated by quantitative real-time PCR and two of them had significant expression change. Bioinformatics analysis was performed to observe the features of hub genes, including hub gene-RBP/TF/miRNA/drug network construction, functional analysis, and protein-protein interaction network. Pearson's correlation analysis was also performed to identify the correlation between hub genes and autophagy genes, among which there were four autophagy genes significantly correlated with hub genes, including ATG4B, ATG3, ATG13, and ULK1.

**Conclusion:**

The results indicated that autophagy might play an essential role in the process and underlying molecular mechanism of primary ovarian insufficiency, which was revealed for the first time and may help to provide a molecular foundation for the development of diagnostic and therapeutic approaches for primary ovarian insufficiency.

## 1. Introduction

Primary ovarian insufficiency (POI), also known as premature menopause or premature ovarian failure (POF), is characterized by cessation of menstruation before the expected age of menopause [1] and is a frequent cause of female infertility. POI is typically defined as at least a 4-month history of oligomenorrhoea and elevated plasma levels of follicle-stimulating hormone (FSH; >25 IU/L) [2]. Numerous complications are found in POI patients, consisting of not only infertility but also sexual dysfunction, vasomotor symptoms, Alzheimer's disease, osteoporosis, and cardiovascular diseases [3]. Respectively, it affects approximately 1 in 100 women at the ages of 40 while 1 in 1000 women at the age of 30 [4].

POI is highly heterogeneous in etiology, such as genetic, autoimmune, iatrogenic, and infectious factors. However, the exact causes of POI remain unknown despite of its strong genetic link, indicating that genes likely to be associated with this condition are yet to be discovered [5]. Currently, the medical treatment of POI is mostly by estrogen supplementation, which has some side effects such as increasing risk of endometrial carcinoma and breast cancer [6]. Hence, it is essential to explore underlying mechanism of POI, which can contribute to elucidate the role of genes in pathogenesis and generate applicable insights to develop preventive strategies, novel diagnostic modalities, and targeted therapeutics. Lipopolysaccharide (LPS) has been revealed to play an essential role in causing hormonal imbalance, ovarian dysfunction, and even infertility [7]. It can be utilized to induce primary ovarian insufficiency (POI) in mouse through causing fibrosis, inflammation, and granulosa cell apoptosis [8]. The exposure to LPS can lead to cell programed death of granulosa cells such as apoptosis and pyroptosis, which contribute to steroidogenesis dysfunction and reproduction failure finally [9]. However, the relationship between LPS-induced POI and autophagy has not been identified.

Autophagy, a lysosomal degradative process of damaged cytoplasmic organelles or cytosolic components, occurs in all eukaryotic cells from yeast to mammals and takes part in numerous physiological processes such as differentiation, development, and aging and contributes to both innate and adaptive immunity [10]. Autophagy was proved to be involved in various diseases based on genetic studies, consisting of inflammatory diseases, neurodegenerative diseases, autoimmune disorders, and cancers. Autophagy could play essential roles in multiple types of tumor through affecting tumor growth, regulating tumorigenesis, giving an adaptive response to cancer cells, and so on [11]. For instance, autophagy participated in breast cancer through regulating tumor immune response [12], involved in uveal melanoma by regulating the expression of Beclin-1 [13, 14], and plays important roles in ovarian cancer through causing cisplatin resistance via mediating ERK [15]. Autophagy is active in cells in a basal state but can also be induced in response to multiple forms of cellular stress, such as nutrient or growth factor deprivation, reactive oxygen species, hypoxia, protein aggregates, DNA damage, damaged organelles, or intracellular pathogens [16]. Basal autophagy is important for maintaining cell function by controlling the quality of proteins and organelles, while under stressful conditions, autophagy plays the principal role to supply nutrients for survival [17]. Macroautophagy starts when a double membrane cisterna envelops cytosolic material, consisting of organelles and proteins, expanding into a vesicle called the autophagosome which then fuses with the endosomal–lysosomal system to form an autolysosome [18]. Multiple autophagy proteins take part in controlling this process [19]. In the ovary, autophagy act as a cell survival mechanism, which is involved in maintaining the endowment of female germ cells prior to establishing primordial follicle pools [20]. Autophagy has also been reported to be involved in follicular atresia, which is cell and developmental stage specific [21].

The studies mentioned above strongly indicated that autophagy variants could also originate primary ovarian insufficiency in human. However, to our knowledge, there is little known about the relationship between POI and autophagy. In our study, we detect the human granulosa cell line (SVOG), which was with or without LPS induced. The expression profiles were analyzed, with an especial focus on the autophagy genes and their function. Moreover, the expression of hub genes was validated by quantitative real-time PCR (qRT-PCR).

## 2. Material and Methods

### 2.1. Study Design

**Figure 1:**
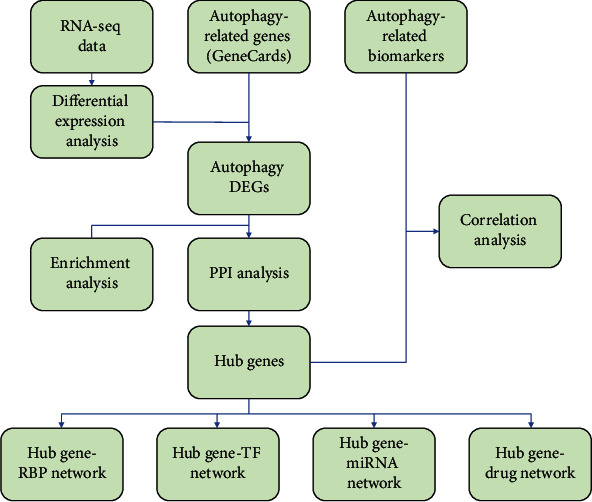
Schematic flow diagram.

In order to illustrate the data preprocessing, analysis, and validation, a schematic flow diagram of the study is presented in Figure 1.

### 2.2. RNA-Sequence Analysis

In our research, the human granulosa cell line (SVOG) was divided into LPS treatment group and normal control group with four samples in each group. The LPS treatment group was performed with LPS, while the normal control group was performed with PBS. Total RNA isolation was performed using RNeasy Plus Mini Kit (QIAGEN), which was followed with RNA-seq. Raw reads in fastq format were analyzed with perl and finally turned into read count of mRNA.

### 2.3. Identification of DEGs

According to the above groups based, we performed normalization and differential gene expression analysis using the “DESeq2” R package. The normalization was based on the “Relative Log Expression” method, which is specifically implemented in the “DESeq2” [22]. The scaling factors were calculated using the median ratio between gene abundances and the geometric mean. As a method for differential analysis of transcriptome count data, DESeq2 improves the interpretability and stability of estimation because of shrinkage estimators for fold change (FC) and dispersion [22]. The differential gene expression analysis was conducted. Then, we specified logFC > 0.5 and *P* value < 0.05 as upregulated genes while logFC < −0.5 and *P* value < 0.05 as downregulated genes, and both upregulated genes and downregulated genes were defined as differentially expressed genes (DEGs). “ggplot2” and “ComplexHeatmap” R package [23] were used to generate the volcano plot and heatmap.

### 2.4. Identification and Functional Analysis of ARGs

Search for the word “autophagy” on the GeneCards (https://www.genecards.org/) to retrieve autophagy-related genes (ARGs), which were downloaded and intersected with DEGs. The intersection was autophagy DEGs.

The GO biological processes were shown in three aspects, including biological processes (BP), cellular components (CC), and molecular functions (MF) [24]. *P* value < 0.05 as the cut of criterion was considered statistically significant [24]. GO enrichment analysis of autophagy DEGs was implemented by clusterProfiler package [25], and items were considered as significantly different if they meet the conditions of *P* value < 0.05 (BH method). R package GOplot [26] was used to integrate the quantitative information by implementing high-quality and novel plotting, which provided us with a collection of multilayered and prespecified charts. Valuable information was added to each layer to display the intended message.

### 2.5. PPI Network Construction and Module Analysis

STRING (https://string-db.org/) is a website about protein interaction, whose aim is to achieve a comprehensive and objective global network and present them with a unique set of computational predictions [27]. In order to explore the mutual relationship between proteins encoded by different genes, autophagy DEGs were imported into STRING website for further analysis. Next, we output the analysis results to a TSV format file and used Cytoscape software (version 3.8.0) for detail processing and module analysis. CytoHubba [28] is a plug-in downloaded from Cytoscape App Store, which can find hub genes in PPI. Therefore, we applied this plug-in to detect top 10 hub genes in PPI network with the process of MMC, DMNC, MNC, Degree, EPC, BottleNeck, EcCentricity, Closeness, Radiality, Betweenness, Stress, and ClusteringCoefficient. After filtering and visualizing with the Upset diagram, STRING website was used again for achieving PPI network of hub genes.

### 2.6. Hub Gene-RBP/TF/miRNA/Drug Network Construction

RNAInter (http://rnainter.org/) provides a more comprehensive and readily accessible RNA interactome platform to investigate the regulatory landscape of cellular RNAs [29]. RNAInter was used to facilitate elucidating the role of RNA-binding protein (RBP), whose score was more than 0.15.

hTFtarget (http://bioinfo.life.hust.edu.cn/hTFtarget) provides a comprehensive, reliable, and user-friendly resource for exploring human TF-target regulations, which is useful for a wide range of users in the transcription factors (TF) and gene expression regulation community [30]. KnockTF (http://www.licpathway.net/KnockTF/index.html) constructs a TF-differentially expressed gene network and performs network analyses for genes of interest, which can help elucidate TF-related functions and potential biological effects [31]. We predicted transcription factors through hTFtarget and KnockTF with the criterion of logFC ≤ −1 and network was constructed according to those TFs.

ENCORI (The Encyclopedia of RNA Interactomes, http://starbase.sysu.edu.cn/index.php) showed extensive and complex RNA–RNA and protein–RNA interaction networks by analyzing a large set of Ago and RBP binding sites derived from all available CLIP-Seq experimental techniques (PAR-CLIP, HITS-CLIP, iCLIP, and CLASH) [32]. The Drug-Gene Interaction Database (DGIdb, http://www.dgidb.org) is a web resource that provides information on drug-gene interactions and druggable genes from publications, databases, and other web-based sources [33]. They were both used to explore the miRNA interaction networks and potential drugs. The results were visualized by Cytoscape.

### 2.7. Delineation of Association between Hub Genes and Autophagy Genes

Autophagy is a lysosomal degradation pathway, which is essential for survival, differentiation, development, and homeostasis. We examine and filter the expression profile, through which we get the autophagy genes: ATG5, ATG16L1, ATG12, ATG13, ULK1, LAMP1, LAMP2, UVRAG, ATG3, ATG4A, ATG4C, ATG4D, ATG4B, ATG7, and ATG10. Pearson's correlation analysis was used between hub genes and autophagy genes mentioned above.

### 2.8. Validation of Autophagy and Expression of Hub Genes

Samples of human granulosa cell line SVOG, consisting of the normal control group and LPS-treated group, were collected and lysed in RIPA lysis buffer (PC101, EpiZyme, China) and then proteins were extracted. The BCA protein assay kit (NCM Biotech, China) was employed to detect the protein concentration. The proteins (15 *μ*g) were loaded and electrophoresed on 12.5% SDS-polyacrylamide gels and then transferred to PVDF membranes. After blocking with protein-free rapid blocking buffer (PS108P, EpiZyme, China) for 30 min at room temperature, the PVDF membranes were probed with primary antibodies against LC3 (1 : 2000, CST) or P62 (1 : 2000, CST) at 4°C overnight. And the anti-*β*-actin antibody (1 : 5000, Abclonal, China) was used as the internal control. Then, the membranes were incubated with secondary antibodies for 90 min at room temperature. The ECL western blotting kit (NCM Biotech, China) was applied to visualize the targeted protein bands. We used the ImageJ software to evaluate the protein band densities.

Total RNA was isolated from the human granulosa cell line SVOG, which was with or without LPS induced, using TRIzol (RNAiso Plus; Takara, Japan) according to the manufacturer's instructions. We also obtained RNA samples from another human granulosa cell line KGN in order to avoid deviation. One microgram of RNA was reversely transcribed into cDNA using a PrimeScript™ RT reagent kit (Takara). Amplification was performed using TB Green® Premix EX Taq™ II (Takara) and gene-specific primers (Sangon, Shanghai, China) on a qRT-PCR device (QuantStudio 5, Thermo Fisher Scientific, Waltham, MA, USA). *β*-Actin was used as an internal control. The relative expression of the genes was calculated using the 2-*ΔΔ*CT method.

### 2.9. Statistical Analysis

The statistical analyses were performed using R 4.0.2 (https://www.r-project.org/, version 3.6.3). All the statistical tests were 2-sided, and *P* < 0.05 was considered statistically significant. In addition, results were analyzed statistically using the Mann-Whitney *U* test and Wilcoxon *t*-test (*P* < 0.05).

## 3. Results

### 3.1. Identification of Differentially Expressed Genes

**Figure 2:**
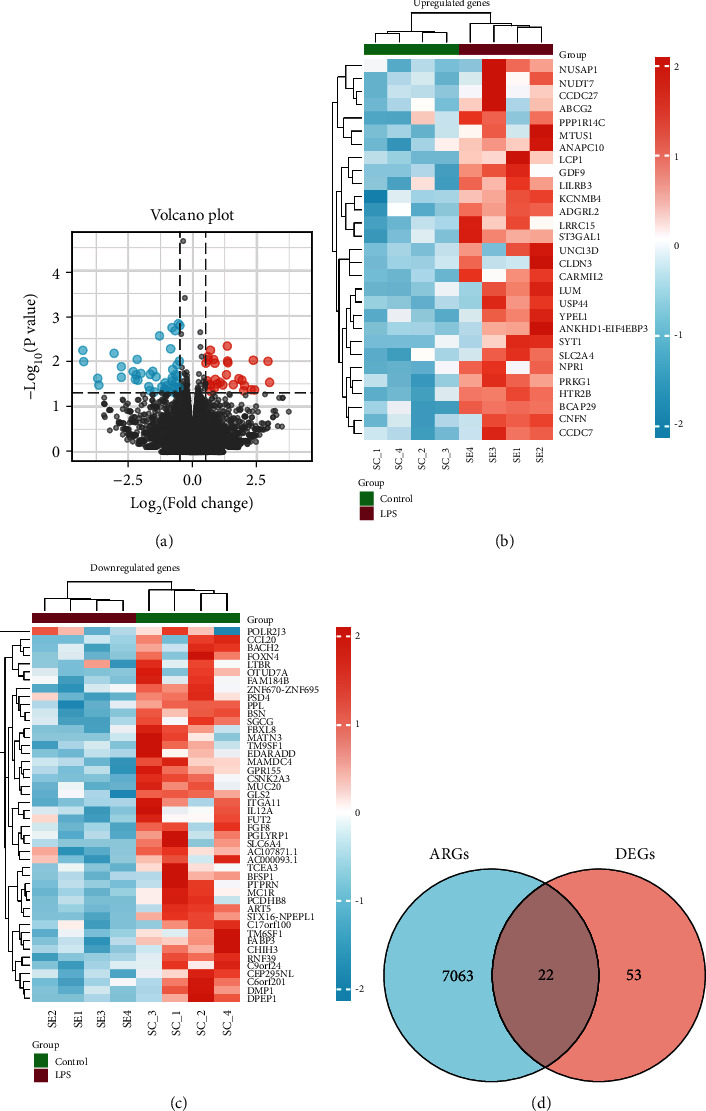
Identification of differentially expressed genes. (a) The differentially expressed genes. (b) Venn diagram of ARGs and DEGs. (c) The upregulated DEGs. (d) The downregulated DEGs.

After standardization of microarray results, the differentially expressed genes (DEGs) were identified, and the results illustrated that there were 29 upregulated genes and 46 downregulated genes (Figures 2(a)–2(c)). Then, we downloaded autophagy genes from GeneCards. The overlap among the autophagy genes and DEGs contained 22 genes as shown in the Venn diagram (Figure 2(d)).

### 3.2. GO Enrichment Analyses of DEGs

**Figure 3:**
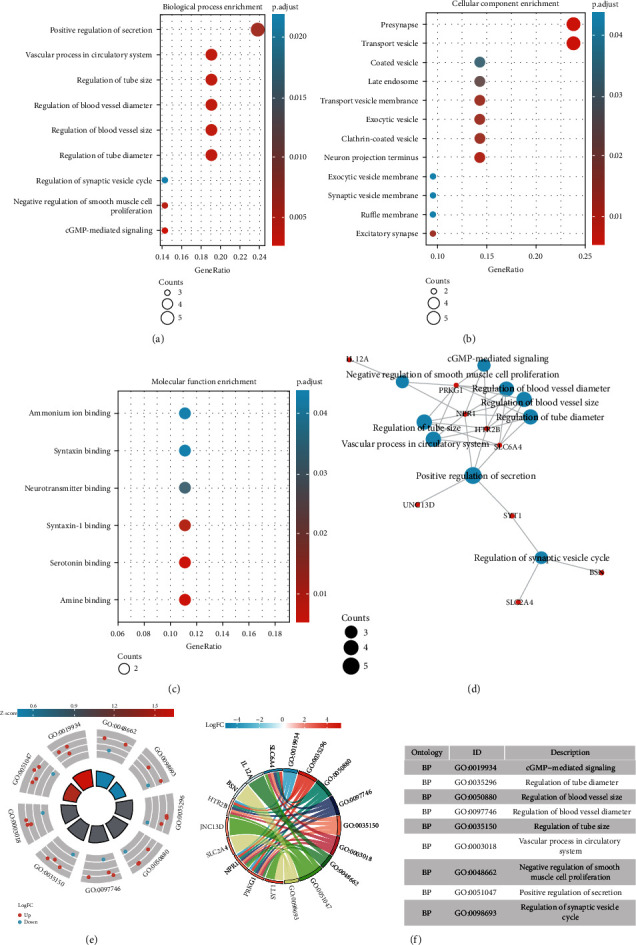
GO enrichment analyses of DEGs. (a) Biological process enrichment by GO analysis. (b) Cellular component enrichment by GO analysis. (c) Molecular function enrichment by GO analysis. (d–f) GO enrichment analysis.

To analyze the biological classification of DEGs, functional and pathway enrichment analyses were performed, and the results are shown in Table 1. GO analysis results showed that changes in biological processes (BP) of DEGs were significantly enriched in cGMP-mediated signaling, regulation of tube diameter, regulation of blood vessel size, regulation of blood vessel diameter, regulation of tube size, vascular process in circulatory system, negative regulation of smooth muscle cell proliferation, positive regulation of secretion, and regulation of synaptic vesicle cycle (Figures 3(a) and 3(d)–3(f)). Changes in cell component (CC) of DEGs were mainly enriched in transport vesicle, presynapse, neuron projection terminus, clathrin-coated vesicle, excitatory synapse, exocytic vesicle, transport vesicle membrane, late endosome, coated vesicle, ruffle membrane, synaptic vesicle membrane, and exocytic vesicle membrane (Figure 3(b)), while changes in molecular function (MF) were mainly enriched in amine binding, serotonin binding, syntaxin-1 binding, neurotransmitter binding, syntaxin binding, and ammonium ion binding (Figure 3(c)).

### 3.3. PPI Network Construction and Hub Gene Selection

**Figure 4:**
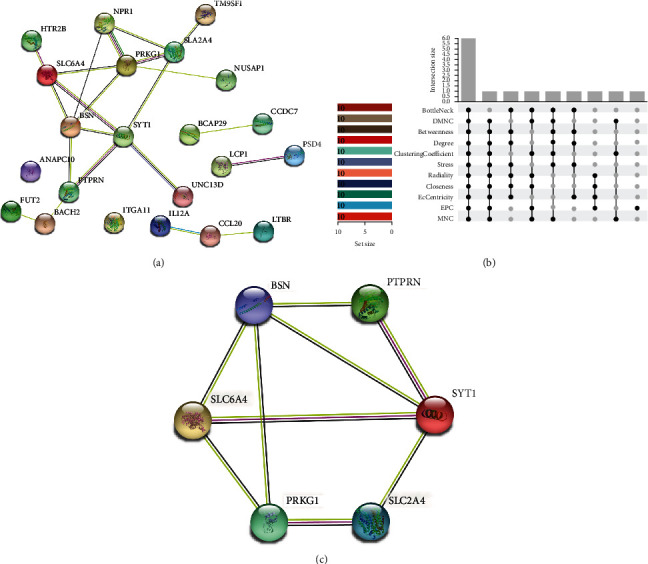
PPI network construction and hub gene selection. (a) PPI network construction. (b) Hub gene selection. (c) PPI network of hub genes.

The PPI network of autophagy DEGs was constructed (Figure 4(a)), which illustrated that there were 22 autophagy DEGs correlated with POI as well as 22 protein-protein interactions. CytoHubba was used to filtrate data for hub genes (Figure 4(b)). A total of 6 genes were identified as hub genes with significance in all 11 arithmetic, which indicated that they might play important role in the process of POI (Table 2).

### 3.4. Construction of Hub Gene-RBP/TF/miRNA/Drug Network

**Figure 5:**
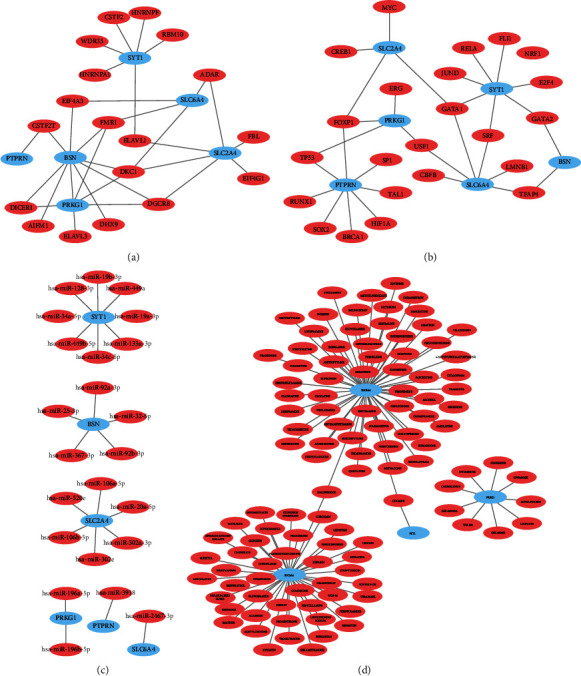
Construction of hub gene-RBP/TF/miRNA/drug network. (a) Construction of hub gene-RBP network. (b) Construction of hub gene-TF network. (c) Construction of hub gene-miRNA network. (d) Construction of hub gene-drug network.

Public databases were used to explore potential RBP, TF, and miRNA and predict potential therapeutic agents, which might possibly interact with hub genes. The results indicated that DKC1 was the most potential gene according to RNAInter database (Figure 5(a)). The hTFtarget database and KnockTF database were used to predict the potential transcription factors (Figure 5(b)). The results revealed that FOXP1 could activate PTPRN, PRKG1, and SLC2A4, while GATA1 was able to activate SLC2A4, SLC6A4, and SYT1. Furthermore, regulatory microRNAs (miRNAs) were predicted for hub genes and used to establish a potential hub gene-miRNA regulation network using the ENCORI platform (Figure 5(c)). We uploaded hub genes to the DGIdb database, and all drug options have been approved, among which there were two kinds of drugs that could target two hub genes simultaneously, namely, HALOPERIDOL and COCAINE. The hub gene-drug network is constructed and shown in Table 3.

### 3.5. Correlation between Hub Genes and Autophagy Genes

**Figure 6:**
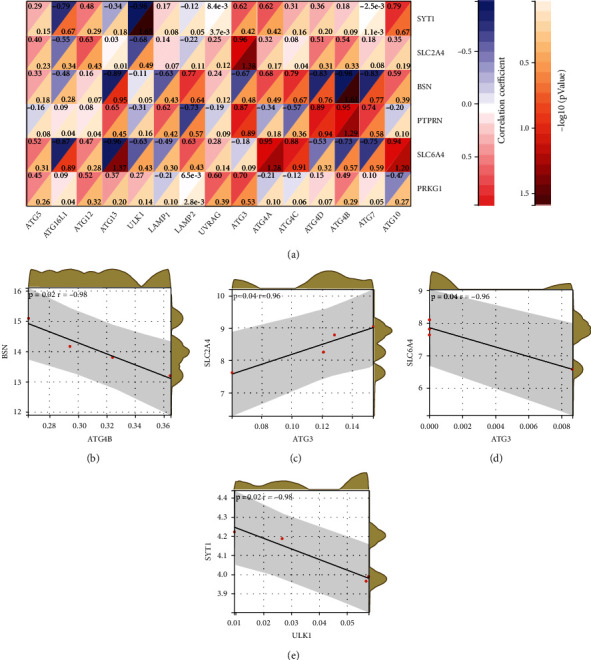
Correlation between hub genes and autophagy genes. (a) Landscape of the correlation between hub genes and autophagy genes. (b) The correlation between BSN and ATG4B. (c) The correlation between SLC2A4 and ATG3. (d) The correlation between SLC6A4 and ATG13. (e) The correlation between SYT1 and ULK1.

To explore the impact of hub genes on POI, we analyzed the correlation between hub genes and autophagy genes by Pearson's correlation (Figure 6(a)). The results indicated that there was notable significant correlation between autophagy genes and some hub genes.

### 3.6. Expression Analysis of Hub Genes and Validation of Autophagy

**Figure 7:**
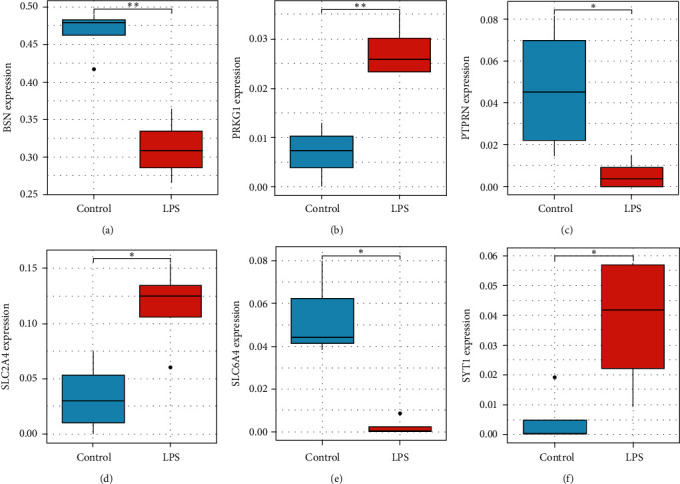
Expression analysis of hub genes. (a–f) The expression level of hub genes.

**Figure 8:**
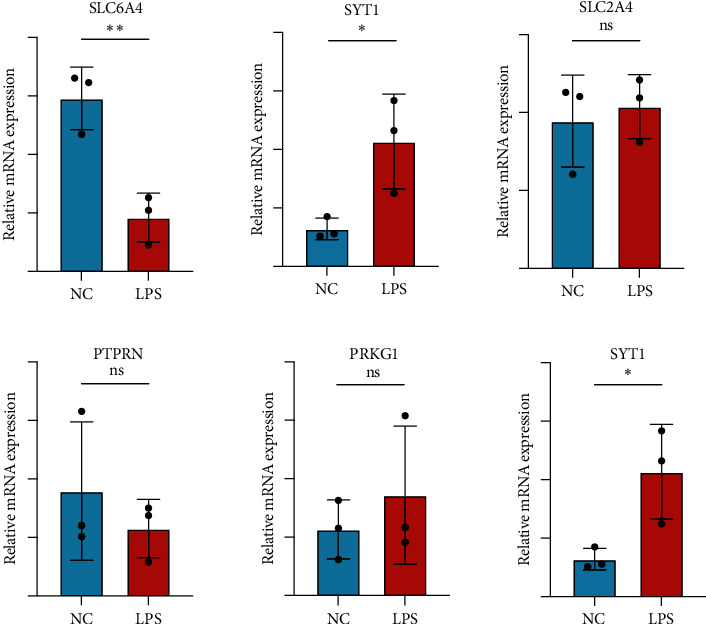
Validation of the expression of hub genes in SVOG.

The differential expression of hub genes was revealed based on FPKM datasets. Compared to normal control, the gene expression changed in LPS-treated group, in which BSN, PTPRN, and SLC6A4 downregulated while PRKG1, SLC2A4, and SYT1 notably upregulated (Figure 7). The results were validated in human granulosa cell lines KGN and SVOG by qRT-PCR, revealing upregulated expression of PRKG1, SLC2A4, and SYT1 and downregulated gene expression of BSN, PTPRN, and SLC6A4, among which the expression changes of SYT1 and SLC6A4 in SVOG cell line were of significance (Figure 8 and Figure S1). The primer sequences of hub genes were shown in Table 4.

**Figure 9:**
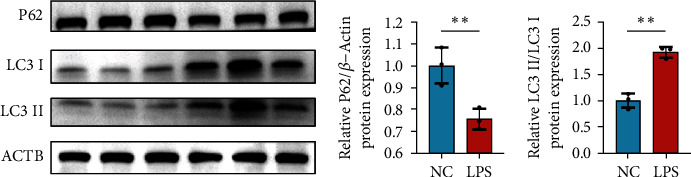
Autophagy promotion after LPS treatment.

After treated with LPS, the expression of proteins related to autophagy significantly changed (Figure 9). The ratio of LC3 II/I increased while the expression of P62 decreased, which both indicated that autophagy was promoted after LPS treatment.

## 4. Discussion

POI is characterized by marked heterogeneity, which is with a significant genetic contribution. However, it is challenging to identify exact causative genes because of numerous unduplicated discoveries. It is essential and necessary to take stock of the field, frame the progress, outline the controversies, and evaluate future directions in elucidating the genetics of POI. Multidisciplinary approaches could help to explore molecular signatures [34], which accounts for the progression of disease and contribute to searching for specific treatment [35]. Identification of effective and novel therapy strategies based on multiomics methods can make it possible to have a better management and improve the prognosis of patients [36]. Hence, we applied various approaches to reveal the underlying mechanism of POI, consisting of bioinformatics, cellular biology, and molecular biology.

In our study, we performed RNA sequence analysis between the LPS-treated group and normal control group, whose results were then used to identify the differentially expressed genes. Functional and pathway enrichment analysis and PPI network were performed to analyze the biological classification of DEGs. According to arithmetic in CytoHubba, six genes were identified as hub genes, namely, BSN, PTPRN, SLC6A4, PRKG1, SLC2A4, and SYT1. The expression of hub genes was revealed based on FPKM datasets as well as validated by qPCR, while various characteristics of hub genes were also identified, such as potential RBP, TF, miRNA, drugs, and correlation with autophagy genes.

Compared to normal control, some hub gene expression downregulated in LPS-treated group, including BSN, PTPRN, and SLC6A4, while some upregulated, consisting of PRKG1, SLC2A4, and SYT1. The expression change was validated through qRT-PCR, which demonstrated the same regulated tendency of bioinformatic analysis and proved the significant upregulated expression of SYT1 and notable downregulated expression of SLC6A4. We also accessed autophagy through identifying P62 and LC3 protein expression. The expression of P62 decreased while LC3 II/I increased after LPS treatment, indicating that LPS promoted autophagy in granulosa cells [37].

SYT1 is an important presynaptic vesicle protein which binds Ca2+ to regulate synaptic vesicle exocytosis and was revealed to act as an important regulator in mouse oocyte activation events consisting of cortical granule exocytosis and the generation of Ca2+ signals [38]. SLC6A4 encodes an integral membrane protein, which act as the role to transport the neurotransmitter serotonin from synaptic spaces into presynaptic neurons. SLC6A4 5HTTLPR polymorphism was revealed to be related to insulin blood levels and insulin secretion during OGTT in patients with polycystic ovary syndrome (PCOS) [39]. Recent research has implied that BSN acted in concert to control presynaptic autophagy [40]. PTPRN encodes a member of the protein tyrosine phosphatase (PTP) family, which is known to be signaling molecules that regulate a variety of cellular processes including cell growth, differentiation, mitotic cycle, and oncogenic transformation. Protein tyrosine phosphatase-1B (PTP1B), encoded by PTPRN, serves as an essential negative regulator for insulin signaling, whose ablation was reported to protect against ER stress-induced cardiac anomalies through regulation of autophagy [41]. Protein kinase cGMP-dependent 1(PRKG1) was involved in cGMP-mediated signaling [42], which was also revealed in the biological process enrichment. The activity of PRKG1 in follicular cells was demonstrated to be essential for oocyte maturation [43] and was proved to play important role in the glucose uptake of granulosa cells mediated by nitric oxide [44]. The expression of SLC2A4 might be suppressed in PCOS women, which may consequently alter endometrial function [45].

To demonstrate whether POI was correlated with autophagy, Pearson's correlation was utilized to explore between hub genes and autophagy genes. It was found that the expression levels of BSN had a high negative correlation with ATG4B and the expression levels of SYT1 also had a high negative correlation with ULK1. High expression levels of SLC2A4 were correlated with high expression levels of ATG3, while high SLC6A4 expression was related with low expression levels of ATG13. Therefore, we demonstrated that there might be various correlations between POI and autophagy, which lead to the notable expression changes of autophagy genes.

For patients with genetic risk of POI, novel methods aiming at fertility preservation can benefit them a lot through early diagnosis. Recent research, for instance, revealed that there was close correlation between POI and the mutations of BRCA [46]. BRCA can bind to various essential regulatory proteins and regulate gene expression [47], which make it possible for BRCA to play a novel role in the regulation of autophagy [48]. The assessment of BRCA mutations could be detected by next-generation sequencing or droplet digital PCR [49]. Novel techniques applied for disease early diagnosis could help patients to get a better management and prognosis. It is important to perform further study to identify the correlation between gene regulation and disease, while it is equally essential to search for and validate the application of novel techniques.

However, there were some limitations in our study. Firstly, the pathogenesis of POI is multidimensional, and it is not convincing enough to demonstrate POI only in the aspect of autophagy. Secondly, more clinical characteristics of POI patients should be included in subgroup analysis and future study. Thirdly, though we validated the expression of hub genes in mRNA expression level through qRT-PCR, it is necessary to validate the protein expression and perform more multicenter and prospective studies to evaluate the possible applications of molecular signatures in the future. In addition, further studies containing in vivo and in vitro experiments are required to elucidate the molecular mechanisms of hub genes for clinical applications.

## 5. Conclusion

In conclusion, we performed RNA sequence analysis to extract the DEGs, based on which we constructed PPI network and identified six hub genes. The expression of hub genes was not only revealed based on FPKM datasets but validated by qPCR, while numerous characteristics of hub genes were also identified, consisting of potential RBP, TF, miRNA, drugs, and relationship with autophagy genes. The results indicated that autophagy might play an essential role in the process and underlying molecular mechanism of POI.

## Figures and Tables

**Table 1:** GO enrichment analysis.

Ontology	ID	Description	Gene ID	Count
BP	GO:0019934	cGMP-mediated signaling	HTR2B/NPR1/PRKG1	3
BP	GO:0035296	Regulation of tube diameter	HTR2B/NPR1/PRKG1/SLC6A4	4
BP	GO:0050880	Regulation of blood vessel size	HTR2B/NPR1/PRKG1/SLC6A4	4
BP	GO:0097746	Regulation of blood vessel diameter	HTR2B/NPR1/PRKG1/SLC6A4	4
BP	GO:0035150	Regulation of tube size	HTR2B/NPR1/PRKG1/SLC6A4	4
BP	GO:0003018	Vascular process in circulatory system	HTR2B/NPR1/PRKG1/SLC6A4	4
BP	GO:0048662	Negative regulation of smooth muscle cell proliferation	IL12A/NPR1/PRKG1	3
BP	GO:0051047	Positive regulation of secretion	HTR2B/NPR1/SLC6A4/SYT1/UNC13D	5
BP	GO:0098693	Regulation of synaptic vesicle cycle	SLC2A4/SYT1/BSN	3
CC	GO:0030133	Transport vesicle	PTPRN/SLC2A4/SYT1/BSN/UNC13D	5
CC	GO:0098793	Presynapse	PTPRN/SLC2A4/SLC6A4/SYT1/BSN	5
CC	GO:0044306	Neuron projection terminus	PTPRN/SYT1/BSN	3
CC	GO:0030136	Clathrin-coated vesicle	SLC2A4/SYT1/UNC13D	3
CC	GO:0060076	Excitatory synapse	SYT1/BSN	2
CC	GO:0070382	Exocytic vesicle	SYT1/BSN/UNC13D	3
CC	GO:0030658	Transport vesicle membrane	PTPRN/SYT1/BSN	3
CC	GO:0005770	Late endosome	IL12A/SLC2A4/UNC13D	3
CC	GO:0030135	Coated vesicle	SLC2A4/SYT1/UNC13D	3
CC	GO:0032587	Ruffle membrane	LCP1/PSD4	2
CC	GO:0030672	Synaptic vesicle membrane	SYT1/BSN	2
CC	GO:0099501	Exocytic vesicle membrane	SYT1/BSN	2
MF	GO:0043176	Amine binding	HTR2B/SLC6A4	2
MF	GO:0051378	Serotonin binding	HTR2B/SLC6A4	2
MF	GO:0017075	Syntaxin-1 binding	SLC6A4/SYT1	2
MF	GO:0042165	Neurotransmitter binding	HTR2B/SLC6A4	2
MF	GO:0019905	Syntaxin binding	SLC6A4/SYT1	2
MF	GO:0070405	Ammonium ion binding	HTR2B/SLC6A4	2

**Table 2:** The list of hub genes.

Gene symbol	Description	logFC	Degree
BSN	Bassoon presynaptic cytomatrix protein	-0.563326995	5
PRKG1	Protein kinase cGMP-dependent 1	2.017667849	5
PTPRN	Protein tyrosine phosphatase receptor type N	-3.054326758	3
SLC2A4	Solute carrier family 2 member 4	1.794934871	4
SLC6A4	Solute carrier family 6 member 4	-4.23050068	4
SYT1	Synaptotagmin 1	2.983723141	5

**Table 3:** Hub gene-drug network.

Gene	Drug	Interaction types	Sources
SYT1	Cocaine		PharmGKB
SLC2A4	Streptozocin		NCI
SLC2A4	Genistein		NCI
SLC2A4	Insulin		NCI
SLC2A4	CHEMBL35482		NCI
SLC2A4	Irbesartan		NCI
SLC2A4	Acetylcysteine		NCI
SLC2A4	Phentolamine		NCI
SLC2A4	Clofibrate		NCI
SLC2A4	Verapamil		NCI
SLC2A4	Sorbitol		NCI
SLC2A4	Nystatin		NCI
SLC2A4	Glufosfamide		TdgClinicalTrial
SLC2A4	Dexamethasone		NCI
SLC2A4	Glyburide		NCI
SLC2A4	Psyllium seed husks		NCI
SLC2A4	Dipyridamole		NCI
SLC2A4	Staurosporine		NCI
SLC2A4	Glipizide		NCI
SLC2A4	Etoposide phosphate		NCI
SLC2A4	Penicillamine		NCI
SLC2A4	UCN-01		NCI
SLC2A4	Fludeoxyglucose-F18		NCI
SLC2A4	Estradiol		NCI
SLC2A4	Indinavir		NCI
SLC2A4	Heparin		NCI
SLC2A4	Neomycin		NCI
SLC2A4	Wortmannin		NCI
SLC2A4	Resveratrol		NCI
SLC2A4	Acarbose		NCI
SLC2A4	Liothyronine sodium		NCI
SLC2A4	Epigallocatechin gallate		NCI
SLC2A4	Prasterone		NCI
SLC2A4	Uridine		NCI
SLC2A4	Mirtazapine		NCI
SLC2A4	Soybean oil		NCI
SLC2A4	Curcumin		NCI
SLC2A4	Imatinib		NCI
SLC2A4	Omapatrilat		NCI
SLC2A4	Progesterone		NCI
SLC2A4	Indomethacin		NCI
SLC2A4	Troglitazone		NCI
SLC2A4	Haloperidol		NCI
SLC2A4	Epinephrine		NCI
SLC2A4	Colchicine		NCI
SLC6A4	Doxepin	Inhibitor	TdgClinicalTrial|TEND|GuideToPharmacology
SLC6A4	Cocaine	Inhibitor	TdgClinicalTrial|TEND
SLC6A4	Amitriptyline	Inhibitor	TdgClinicalTrial|TEND|GuideToPharmacology
SLC6A4	Levomilnacipran	Inhibitor	TdgClinicalTrial|GuideToPharmacology
SLC6A4	Sibutramine	Inhibitor	TdgClinicalTrial|TEND|GuideToPharmacology
SLC6A4	Dapoxetine	Inhibitor	TdgClinicalTrial|GuideToPharmacology
SLC6A4	Fluvoxamine	Inhibitor	TdgClinicalTrial|TEND|GuideToPharmacology
SLC6A4	Protriptyline	Inhibitor	TdgClinicalTrial|TEND|GuideToPharmacology
SLC6A4	Sertraline	Inhibitor|binder|negative modulator	TdgClinicalTrial|TEND|GuideToPharmacology
SLC6A4	Ziprasidone	Inhibitor	GuideToPharmacology
SLC6A4	Nortriptyline	Inhibitor	TdgClinicalTrial|TEND|GuideToPharmacology
SLC6A4	Atomoxetine	Inhibitor|binder	GuideToPharmacology
SLC6A4	Amoxapine	Inhibitor	TdgClinicalTrial|TEND|GuideToPharmacology
SLC6A4	Trimipramine	Inhibitor	TdgClinicalTrial|TEND|GuideToPharmacology
SLC6A4	Dexfenfluramine	Inhibitor	TdgClinicalTrial|TEND
SLC6A4	Nefazodone	Inhibitor	TdgClinicalTrial|TEND|GuideToPharmacology
SLC6A4	Phentermine	Inhibitor	TdgClinicalTrial|TEND
SLC6A4	Milnacipran	Inhibitor	TdgClinicalTrial|TEND|GuideToPharmacology
SLC6A4	Lofepramine	Inhibitor	GuideToPharmacology
SLC6A4	Vortioxetine	Inhibitor	TdgClinicalTrial|GuideToPharmacology
SLC6A4	Trazodone	Inhibitor	TdgClinicalTrial|TEND
SLC6A4	Methylphenidate	Inhibitor	TdgClinicalTrial|TEND
SLC6A4	Citalopram	Inhibitor	TdgClinicalTrial|TEND|GuideToPharmacology
SLC6A4	Zotepine	Inhibitor|antagonist	GuideToPharmacology
SLC6A4	Vilazodone	Inhibitor	GuideToPharmacology
SLC6A4	Phenelzine	Inhibitor	GuideToPharmacology
SLC6A4	Imipramine	Inhibitor	TdgClinicalTrial|TEND|GuideToPharmacology
SLC6A4	Duloxetine	Inhibitor	TdgClinicalTrial|TEND|GuideToPharmacology
SLC6A4	Desvenlafaxine	Inhibitor	TdgClinicalTrial|TEND|GuideToPharmacology
SLC6A4	Venlafaxine	Inhibitor	TdgClinicalTrial|TEND|GuideToPharmacology
SLC6A4	Desipramine	Inhibitor	TdgClinicalTrial|TEND|GuideToPharmacology
SLC6A4	Dothiepin	Inhibitor	GuideToPharmacology
SLC6A4	Fluoxetine	Inhibitor	TdgClinicalTrial|NCI|TEND|GuideToPharmacology
SLC6A4	Lumateperone	Inhibitor	TdgClinicalTrial|GuideToPharmacology
SLC6A4	Clomipramine	Inhibitor	TdgClinicalTrial|TEND|GuideToPharmacology|PharmGKB
SLC6A4	Pseudoephedrine	Inhibitor	TdgClinicalTrial
SLC6A4	Methamphetamine	Negative modulator	TdgClinicalTrial
SLC6A4	Tramadol	Inhibitor	TdgClinicalTrial|TEND
SLC6A4	Minaprine	Inhibitor	TdgClinicalTrial|TEND
SLC6A4	Paroxetine	Inhibitor	TdgClinicalTrial|TEND|GuideToPharmacology
SLC6A4	Escitalopram	Inhibitor	TdgClinicalTrial|TEND|GuideToPharmacology
SLC6A4	Solriamfetol		TdgClinicalTrial
SLC6A4	4-Methylthioamphetamine		DTC
SLC6A4	Olanzapine		PharmGKB
SLC6A4	Tedatioxetine		TdgClinicalTrial
SLC6A4	Ribavirin		PharmGKB
SLC6A4	Ondansetron		PharmGKB
SLC6A4	Quetiapine		PharmGKB
SLC6A4	Haloperidol		PharmGKB
SLC6A4	Morphine		PharmGKB
SLC6A4	Bupropion		PharmGKB
SLC6A4	Methadone		PharmGKB
SLC6A4	Tesofensine		TdgClinicalTrial
SLC6A4	Evodiamine		PharmGKB
SLC6A4	Clozapine		PharmGKB
SLC6A4	Berberine		PharmGKB
SLC6A4	Alcohol		PharmGKB
SLC6A4	Buprenorphine		PharmGKB
SLC6A4	Risperidone		PharmGKB
PRKG1	GSK-690693	Inhibitor	GuideToPharmacology
PRKG1	Ipatasertib	Inhibitor	GuideToPharmacology
PRKG1	GSK-269962A		DTC
PRKG1	CHEMBL225519		DTC
PRKG1	Linifanib		DTC
PRKG1	Sotrastaurin		DTC
PRKG1	Cenisertib		DTC
PRKG1	GW843682X		DTC
PRKG1	TAE-684		DTC

**Table 4:** The primer sequences of hub genes.

	Forward primer sequence (5′→3′)	Reverse primer sequence (5′→3′)
SYT1	AAAGTCCACCGAAAAACCCTT	CCACCCAATTCCGAGTATGGT
PRKG1	GGACAGGACTCATCAAGCATAC	CTTCACGAGTGACATTTACCGTT
SLC6A4	TGACACACGGCACTCTATCC	AGCCAATCACTGAGAGAAGGA
PTPRN	TTGAGCATGACCCTCGGATG	GCCAGAAGTCTGCGATGGTAT
BSN	GCCCTCTATCCACCAAGGC	GTCTTGCTGGGTTCAGAAGC
SLC2A4	GCCATGAGCTACGTCTCCATT	GGCCACGATGAACCAAGGAA

## Data Availability

The data used to support the findings of this study are available from the corresponding author upon request.

## Supplementary Materials

Supplementary Materials Figure S1: validation of the expression of hub genes in KGN.
