# Supplementary figures and images for: Identification and Validation of Autophagy-Related Genes in Primary Ovarian Insufficiency by Gene Expression Profile and Bioinformatic Analysis
Source: Anal Cell Pathol (Amst). 2022 Jul 4;2022:9042380. doi: 10.1155/2022/9042380 (PMC9273469; doi:10.1155/2022/9042380)

**SLC6A4**

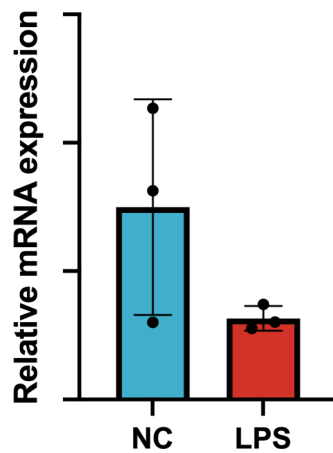

**SYT1**

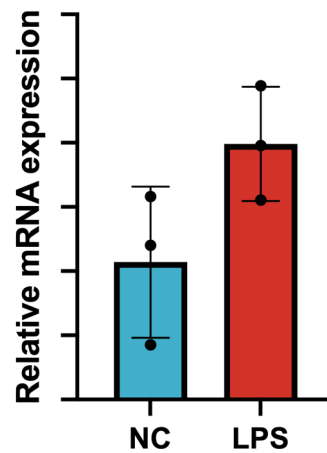

**SLC2A4**

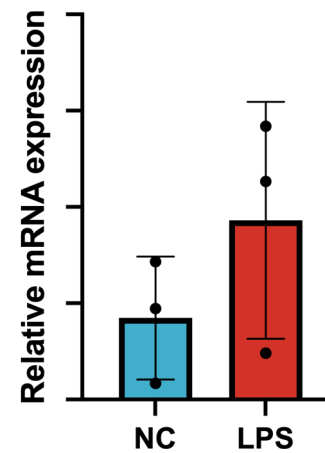

**PTPRN**

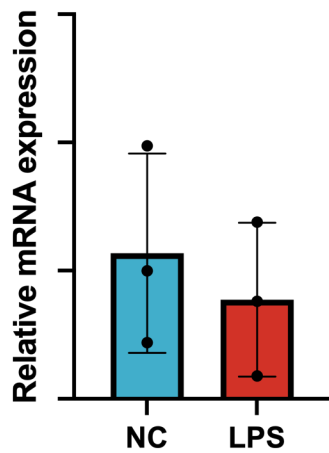

**BSN**

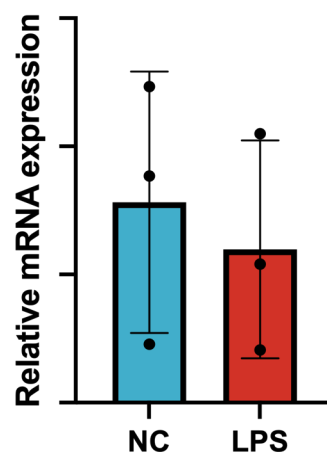

**PRKG1**

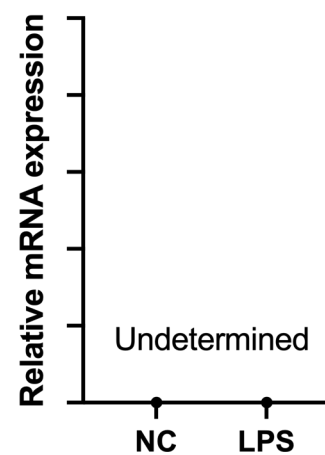

Supplement: Supplementary Materials — Figure S1: validation of the expression of hub genes in KGN. [file 9042380.f1.pdf]
